# Lw-CNN-Based Myoelectric Signal Recognition and Real-Time Control of Robotic Arm for Upper-Limb Rehabilitation

**DOI:** 10.1155/2020/8846021

**Published:** 2020-12-28

**Authors:** Benzhen Guo, Yanli Ma, Jingjing Yang, Zhihui Wang, Xiao Zhang

**Affiliations:** ^1^College of Information Science and Engineering, Hebei North University, Zhang Jiakou 075000, China; ^2^Population Health Informatization in Hebei Province Engineering Technology Research Center, Zhang Jiakou 075000, China

## Abstract

Deep-learning models can realize the feature extraction and advanced abstraction of raw myoelectric signals without necessitating manual selection. Raw surface myoelectric signals are processed with a deep model in this study to investigate the feasibility of recognizing upper-limb motion intents and real-time control of auxiliary equipment for upper-limb rehabilitation training. Surface myoelectric signals are collected on six motions of eight subjects' upper limbs. A light-weight convolutional neural network (Lw-CNN) and support vector machine (SVM) model are designed for myoelectric signal pattern recognition. The offline and online performance of the two models are then compared. The average accuracy is (90 ± 5)% for the Lw-CNN and (82.5 ± 3.5)% for the SVM in offline testing of all subjects, which prevails over (84 ± 6)% for the online Lw-CNN and (79 ± 4)% for SVM. The robotic arm control accuracy is (88.5 ± 5.5)%. Significance analysis shows no significant correlation (*p* = 0.056) among real-time control, offline testing, and online testing. The Lw-CNN model performs well in the recognition of upper-limb motion intents and can realize real-time control of a commercial robotic arm.

## 1. Introduction

Upper-limb rehabilitation robots are an innovative approach to rehabilitation training which lend strength and promote recovery of the upper-limb motion functions in stroke sufferers without over-burdening medical personnel [1–3]. Many upper-limb rehabilitation robots have been developed in recent years. MIT-Manus [4], for example, can help stroke patients regain steady motion capability. MIME [5], T-WREX [6], and NEREBOT [7] can train upper-limb rehabilitation training motions over three degrees of freedom (3DOF). Previous researchers [8] proposed an exoskeleton robot also capable of 3DOF.

Training that is “active” rather than “passive” (i.e., that centers on the patients' intended motions throughout a session rather than forcing them into a set regimen that does not individually vary) can significantly enhance the effects of training and improve patients' rehabilitation experiences [9–11]. Currently, existing man-machine interactive interfaces for body motion intent recognition function are based on three modes: mechanical sensor signals [8], surface myoelectric (sEMG) signals [12], and biological EEG signals [13–15]. Mechanical sensors are accurate and reliable, but they only reflect lag motion information and thus are not conducive to real-time control. Man-machine interfaces based on sEMG signal processing have seen rapid and extensive advancements in recent years. Both sEMG signals and EEG signals reflect human motion intents. Non-instructive surface EMG technology effectively records the electric activities of muscles [16–18]. Unlike the “on-off” [19]or proportional control [16] strategies of traditional rehabilitation robots, patients' sEMG signals are collected as their upper-limb motion intents are acquired via pattern recognition. This allows for natural and flexible interactions between the patient and the rehabilitation robot.

The support vector machine (SVM) [19], LDA [20], and Gaussian mixture models (GMM) [18] are widely applied for classification of robot-acquired signals. The performance of the technologies discussed above greatly depends on the feature selection of signals. In most cases, feature selection is conducted by researchers manually based on their professional experience, which is known as “feature engineering” [21–23]. Deep learning is a machine-learning method which has become a wildly popular research topic in recent years. It does not require manual feature extraction-feature extraction and advanced abstraction can instead be conducted automatically on raw signals [24, 25].

High-performance deep-learning methods such as the convolutional neural network (CNN) have been tested for various gesture recognition applications [26–28]. Existing sEMG signal collection technologies include sparse multichannel sEMG and high-density sEMG (HD-sEMG). HD-sEMG records both temporal and spatial changes in muscle activities via electrode arrays [29]; it can classify as many as eight distinct hand motions [30]. However, an increase in collection channels creates a dramatic increase in the computational burden, which results in an overly complex system that is incapable of real-time upper-limb rehabilitation performance. The sparse multichannel sEMG can recognize upper-limb motion intents while consuming fewer computational resources.

Many previous researchers have explored gesture recognition with deep-learning methods and various dexterous hand and artificial limb applications, but there have been relatively few studies on deep-learning upper-limb rehabilitation robotics technologies. Previous researchers [31, 32] used the traditional SVM algorithm for upper-limb motion intent recognition and applied it in upper-limb rehabilitation robots. Others [33] used a Back Propagation (BP) neural network as a classification model, but this required manual selection of energy and max values as inputs.

Many deep-learning models with different architectures have been proposed for sEMG signal recognition based on deep learning [34–37]. Training and verification are generally carried out on public or self-built datasets in an offline manner. Accuracy usually differs between online and offline recognition [38]; online recognition accuracy is lower than offline [32, 38–40]. High online recognition accuracy and good real-time performance are of great significance in terms of practical application in rehabilitation robots or artificial limbs. Researchers [41] have used Gaussian Naive Bayes (GNB) and SVM for myoelectric signal recognition; their models were verified both online and offline to realize the real-time control of a hand exoskeleton. Others [42] proposed an upper-limb prosthetic real-time control method based on the motor unit drive.

This study was conducted to test the feasibility of a multiple-DOF, real-time robotic arm using myoelectric pattern recognition for upper-limb rehabilitation training. A three-channel sparse electrode is used to collect raw sEMG signals of the deltoid, biceps brachii, and triceps brachii from an upper limb. The signals are then input into a Lw-CNN model for body motion intent recognition. Six rehabilitation motions were designed over the shoulder and elbow joints; then, a dataset was established based on the motions of eight volunteers. The offline trained model was deployed and verified through online recognition. A commercial robotic arm was also tested to preliminarily validate the real-time control performance of the proposed deep-learning model. The entire control course took 269 ms, satisfying the requirements for real-time control within 300 ms [43–46].

## 2. Materials and Methods

### 2.1. Subjects

Eight subjects (denoted *S*1–*S*8) participated in this experiment (Table 1). All the subjects were students of Hebei North University at the time of their participation. All completed a physical examination at the First Affiliated Hospital of Hebei North University and were issued health certificates before joining the experiment. They also signed a consent form to publish details and/or images.

### 2.2. Experimental Protocols

**Figure 1:**
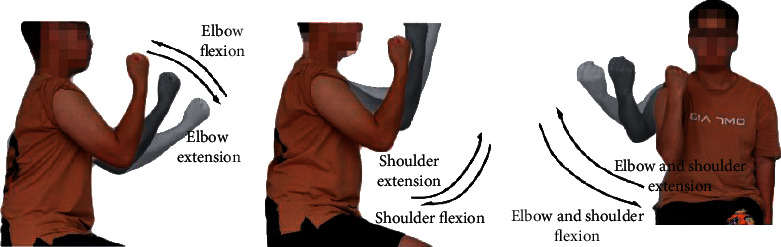
Upper-limb motions. (a) Elbow flexion and extension; (b) shoulder flexion and extension; (c) elbow and shoulder flexion and extension.

The upper-limb rehabilitation robot investigated in this study was designed to train certain motions in stroke patients' elbow joints and shoulder joints. As shown in Figure 1, six motion modes including elbow flexion (EF), elbow extension (EE), shoulder flexion (SF), shoulder extension (SE), elbow & shoulder flexion (ESF), and elbow & shoulder extension (ESE) were designed accordingly.

During his or her interaction with the robot, the subject sat on a chair close to the table with the palm making a fist facing upwards, the forearm perpendicular with the upper arm, and the upper arm forming about a 20° angle with the body for the initial training posture, “EE,” as the upper arm was kept still. The motion of the forearm to the body side from EE is defined as “EF.” From this initial state, the shoulder joints were controlled to move as the upper arm is lifted for “SE.” For the “SF” motion, the upper arm fell from SE back to the initial posture. The “ESE” motion was defined by lifting the upper arm and straightening the forearm from the initial state. EF and EE only concerned motions of the one-degree-of-freedom elbow joint. SF and SE concerned simultaneous motions of elbow and shoulder joints. Subjects actively exerted forces in performing ESF and ESE to control the shoulder and elbow joints simultaneously. These two motions were compounds of the aforesaid four motions, including the abduction motion of upper arms, and involved in the multi-role of musculus biceps brachii, musculus triceps brachii, and deltoid. These six actions were presented as separate action types for identification.

**Figure 2:**
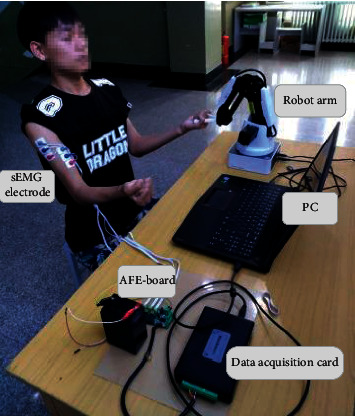
Experimental setup.

Figure 2 shows the experiment setup. Three channels of sEMG signals were collected in this research. sEMG electrodes were, respectively, pasted on the surfaces of musculus biceps brachii, musculus triceps brachii, and deltoid. In the actual control experiment, to ensure safety, the subject's left hand did not touch the tail end of the robotic arm at any point. The system consists of three components: the sEMG acquisition system (EAS), the motion recognition program (MRP), and the robotic arm. As the subject's upper limb moved, sEMG signals were acquired from the activated muscles via EAS. The raw sEMG signals were decoded by the MRP to extract the subject's motion intents, and then the information was sent to the robotic arm.

Each experiment consisted of three sessions: offline myoelectric pattern recognition analysis, online myoelectric pattern recognition analysis, and real-time control sessions. In the first session, sEMG data were recorded to train the deep model while collected samples were used for offline analysis. In the online analysis session, the subject's sEMG signals were collected in real time and sent to the deployed deep model for motion intent recognition. In the third session, the robotic arm was controlled in real time based on the motion intent recognition results of the second session to simulate pulling motions in the subject's “impaired” part to complete the rehabilitation training process.

#### 2.2.1. Session 1: Offline Analysis

In the first session, the robotic arms were powered off while the EAS and MRP started working. Before the experiment, the subjects familiarized themselves with the six motion modes. After starting the experiment, the laboratory technician issued EF, SE, or ESE motion commands to the subjects who then completed motions at a constant rate within 2s according to the instructions received. After 3s, the subjects completed the corresponding extension or flexion motions within 2s and returned to the initial state. EF, SE, and ESE motion sequences were not strictly prescribed. The reason why the definite executive sequences were not used was that the robustness of the model should be verified. Furthermore, other potential factors introduced by fixed sequences on impact test results could be prevented. For example, subjects would subconsciously adapt to principles of such sequences but would unconsciously adjust the strength of motions to enhance the model's recognition accuracy. After repeating the motion 15 times, the subject was given a 5-minute break to combat any muscle fatigue. While completing each motion, the subject applied a level of comfortable force (ranging within 30–40% as a maximum contraction). Three hundred motions were collected from each subject with 50 patterns for each motion. These 300 motions were used to make deep-learning datasets, 70% of which were taken as deep-model training sets and the remaining 30% as testing sets for offline analysis of the deep model.

#### 2.2.2. Session 2: Online Analysis

In this session, sEMG signals were collected and sent to the trained deep model from Session 1 for motion intent recognition. The robotic arm was then powered off. The recognition results and motion sequence numbers were recorded automatically in the program. The subject and tester were not allowed to check the recognition results while the experiment was in progress. Before making each motion, the subject reported the name of the motion to be made to the tester who recorded it immediately. The subject completed the motions in the same manner as in Session 1, with a 5-minute break between every 15 motions. Each motion was completed at least 50 times. The first 50 recognition results of each motion were used for online analysis.

#### 2.2.3. Session 3: Real-Time Robotic Arm Control

In the third session, the robotic arm was powered on. The deep model trained in Session 1 was used to recognize the subject's motion intents; then, motion instructions were sent to the robotic arm in real time. The subject thus received real-time classification feedback. The real-time control experiments, which are based on the online experiments, were additionally provided with the robotic arm control function, in order to verify the delay of the robotic arm system and the data transmission and to observe whether the total delay of the system affected the user experience. Only the CNN classifier was applied in the real-time control experiment because the robotic arms in this experiment should move in accordance with the recognition results. If two classifiers are applied simultaneously, contradictory motion commands might be generated, while the robotic arms only could respond to the motion commands of one classifier. The robotic arm was placed on the left side of the subject and simulated pulling of the patient's left hand to complete the rehabilitation motion of the left upper limb. The subject randomly started performing EF, SE, and ESE motions with the corresponding extension or flexion. Before beginning each motion, the subject reported the motion name to the tester who recorded it alongside the actual motions of the robotic arm. Each motion was completed 50 times. The PC application program recorded the computation duration of the deep model and order-sending delay as a reflection of the system's real-time performance. The time delay of the robotic arm system was determined by checking a datasheet of the robotic arm (10 ms).

### 2.3. Robotic Arm

A robotic arm, the Dobot Magician (Yue Jiang Technology Co. Ltd., Shenzhen, China), was employed in this study. The robotic arm simulated a rehabilitation robot to pull the subject's upper limb to complete the desired motion in real time. Dobot Magician robotic arms are lightweight, easy to program, safe, and capable of 4DOF (3DOF without the end effector) by rotation of the base by −90°–+90°, big arm pitching of 0°–105°, forearm flexion or extension of −10°–−95°, and horizontal rotation of wrist of −90°–+90°. Three stepping motors motivated each DOF through real-time USB-UART connection according to commands given by the computer. The robotic arm's end effector was not installed because the focus of this work is rehabilitation of the shoulder and elbow joints. Three shafts of the robotic arm were moved to simulate pulling of the patient's hand, driving the “impaired” limb to move.

In Session 3 of the experiment, the robotic arm was placed on the left side of the subject with its tail end in horizontal alignment with the palm center under the subject's initial motion (EE), but not touching it. The robotic arm started a robot teaching mode immediately after it was powered on. The tester cooperated with the subject to complete robot teaching operations of the six training motions. The robotic arm was switched into real-time control mode and relevant motions were completed after receiving control instructions sent by the PC.

### 2.4. sEMG Data Acquisition

Six elbow and shoulder joint motions related to the biceps, triceps, and front deltoids were designed to support this experiment. A sparse multichannel sEMG (3 channels) was used to collect sEMG signals reflective of the subjects' upper-limb motion intents. The sEMG acquisition system (EAS) is composed of electrode plates, an analog front-end circuit board (AFE-board) (sAFE-300, Zhituo Intelligent Technology Co. Ltd., Qingdao, China), and USB data collection card (USB3100, ART Technology Co. Ltd., Beijing, China). The subject's skin was cleaned with alcohol before pasting on the electrode plate adjacent to the selected muscle. To simulate a compact electrode, reference electrodes were placed at the non-motorized muscle tendons near the differential electrodes. The raw sEMG signals were very weak in this case and highly susceptible to electromagnetic interference, so they were amplified and filtered with the AFE-board for AD conversion into digital quantities.

The AFE-board is mainly constituted of a differential amplification circuit with AD8220 and a band pass filtering circuit with OPA 364. Raw sEMG signals were input into the data collection card after 1000-multiple differential amplification and 30–400 HZ band pass filtering. The USB3100 data collection card has an 8-channel, 12-digit AD input, 4K FIFO memory, USB2.0 interface with a sampling rate of up to 20 KS/s. The data collection card provides a uniform, shared driving program interface and supports multiple development languages (e.g., Visual C++, NI LabVIEW). The data collection card was configured with the sampling rate of 20 KHz; then, signals were imported into a desktop via USB interface (Windows 10, Intel core i5-7300HQ at 2.5 GHz, NVIDIA GeForce GTX 1050 with 2 GB GDDR5). An application program was developed based on Microsoft Visual C++ and Python3.6 and deployed on the desktop to manage the sEMG data. This program also processed sEMG data with the deep model and controlled the robotic arm that connected to the desktop.

### 2.5. sEMG Data Processing

**Figure 3:**
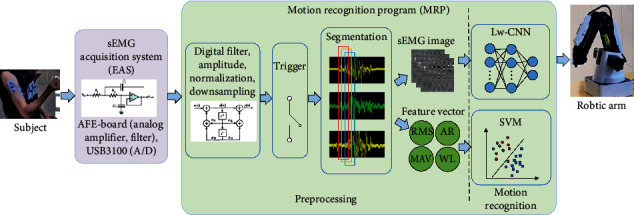
Flowchart of collection and processing of sEMG signals. Note: EAS includes an analog front end filtering amplification circuit and a data collection car. The motion recognition program (MRP) runs on the PC.

Figure 3 shows a diagram of the data collection and processing program, where the collected data is preprocessed before sEMG images and feature vectors are generated and fed into the classifiers for motion intent recognition. In Session 3, in order to avoid different classification results generated from any two classifiers and subsequent contradictory motion instructions for the robotic arm, only the Lw-CNN classifier was used to output results and generate control instructions.

Lw-CNN and SVM models were built based on Tensorflow1.12, an open source machine-learning framework launched by Google. The designed and trained Lw-CNN model can be easily deployed in an embedded system as per its development in Tensorflow.

#### 2.5.1. sEMG Data Preprocessing

The discrete sEMG signals sent by the data collection card were preprocessed for better signal quality and acquisition of inputs needed by the deep model classifier and SVM classifier. As shown in Figure 2, 1 KHz downsampling was conducted on the raw input sEMG signals. A notch filter (*Q* = 30) was established to filter 50 Hz power interference of the AC power supply. A 20–450 Hz digital band pass filter and amplitude normalization were operated to improve the signal-to-noise ratio (SNR) of the sEMG signal. In view of the real-time application of the proposed method, the total duration for collection, preprocessing, classification, and robotic arm control of sEMG signals was restricted within an upper limit of 300 ms [43–46]. The sEMG data were segmented into 192 ms analysis windows with 15 ms of slippage rate including offline/online classification and real-time control.

A motion trigger was used to judge whether a motion had taken place in the experiment. The average mean absolute value (MAV) of each channel signal was computed at the pre-collection stage, wherein 75% of MAV was set as the threshold [47]. The MAV of each analysis window was computed during the experiment. If the threshold was exceeded, the analysis window was deemed to contain motion intent information. The analysis window and two subsequent windows were processed and input into the deep model classifier and SVM classifier for motion intent classification. Three-channel analysis windows containing motion intent information and 192 × 3 = 576 data points were obtained. Three sEMG images were input into the deep model for processing; then, the final classification result was obtained with the majority voting algorithm based on three judgments.

As with most traditional pattern recognition algorithms, the SVM classifier requires a discretized characterization of the signal [32]. In a previous study, different signal eigenvalues were selected in order to train three SVM classifiers for the offline test. Specifically, SVM1 chose the fourth-order autoregressive (AR), the root mean square amplitude (RMS), the waveform length (WL), and the number of zero crossings (ZC) as feature vectors [39]. SVM2 chose the mean absolute value (MAV), the number of zero crossings (ZC), the waveform length (WL), and the number of slope sign changes (SSC) as feature vectors. SVM3 chose the root mean square (RMS) amplitude, the mean absolute value (MAV), the fourth-order autoregressive(AR) coefficients, and waveform length (WL) as feature vectors. The research results proved that the SVM3 classifier boasted the highest recognition accuracy, namely, (82.5 ± 3.5)%. Therefore, the SVM3 classifier was selected for comparison with the Lw-CNN classifier in subsequent experiments.

#### 2.5.2. Deep-Learning Model

**Figure 4:**
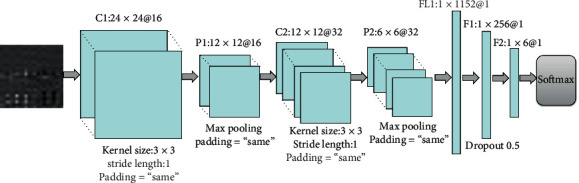
Proposed Lw-CNN architecture.

The feasibility and real-time performance of the upper-limb motion intent recognition based on the Lw-CNN architecture were assessed in a series of experiments. The network topology is shown in Figure 4. The model stacks 2 convolutional blocks composed of one convolutional layer (16 filters 3 × 3 in size, step length of 1, and padding parameter set as “same”) and another convolutional layer (32 filters 3 × 3 in size, step length of 1, and padding parameter set as “same”). A Maxpolling pooling operation (2 × 2 kernel, padding parameter set as “same”) was conducted after each convolution to minimize the quantity of model parameters and prevent overfitting.

After the convolution, two-dimensional sensors were converted into one-dimensional vectors by flattening. A Softmax operation and two fully connected (FC) layers were then imposed. A dropout with a probability of 0.5 was placed after the first fully connected layers to prevent overfitting. All layers have ReLU nonlinearity as activation function and are equipped with Batch-Normalization (BN) to counter the internal covariate shift. The Lw-CNN model presented in this paper can process a 192 ms input window using only 367 k parameters. It is especially well-suited to real-time applications and can be deployed in an embedded system.

### 2.6. Statistical Analysis

The accuracy was computed for each subject under different sessions and with two different recognition models, the Lw-CNN and SVM, to assess the performance of the proposed model. Two-way ANOVA was used to test two classifiers (CNN and SVM) and classification accuracies to compare offline versus online testing (significance level of *p* < 0.05). One-way ANOVA was used to further compare offline and online testing and determine whether there was significant difference when Lw-CNN was used (*p* < 0.05). Confusion matrices of the recognition result with different models under each session were drawn. Precision, recall, and F1-score results were used to evaluate the recognition performance of the model towards the six motions (equations (1)–(3)). FP denotes the number of false positives, TP denotes the number of true positives (labeled correctly), and FN denotes the number of false negatives.

(1) $$\begin{array}{c} \text{precision}=\frac{\text{TP}}{\text{TP}+\text{FP}}, \end{array}$$

(2) $$\begin{array}{c} \text{recall}=\frac{\text{TP}}{\text{TP}+\text{FN}}, \end{array}$$

(3) $$\begin{array}{c} F1‐\text{score}=\frac{2*\text{precision}*\text{recall}}{\text{precision}+\text{recall}}. \end{array}$$

## 3. Results

**Figure 5:**
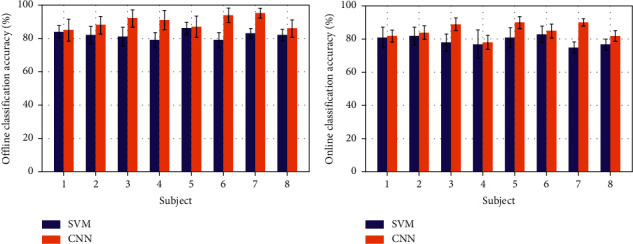
Offline (a) and online (b) classification accuracies of each subject under classifiers L-CNN and SVM. Note: each bar has an error line which denotes the mean value and standard deviation of recognition accuracy across each subject's six respective motions.

Figure 5 shows average classification accuracy of each subject with two classifiers. The error line denotes the standard deviation for recognition accuracy of each subject's six motions. There were significant differences among subjects, but the Lw-CNN model outperformed the SVM model in terms of overall recognition accuracy. The highest recognition accuracy of the Lw-CNN model in offline testing was 95% (*S*7) and the lowest accuracy was 85% (*S*1). The maximum recognition rate of the SVM model was 86% (*S*5) and its lowest rate was 79% (*S*4, *S*6). The standard deviations of recognition accuracy on the motions were lower than 7 for both models with a maximum of 6.5 (*S*1, Lw-CNN). The highest recognition accuracy of Lw-CNN model in online testing was 90% (*S*5, *S*7) and the lowest was 78% (*S*4). The highest recognition rate of the SVM model was 83% (*S*6) and the lowest was 75% (*S*7). The standard deviations of recognition accuracy for the six motions were lower than 7 in both models with a maximum of 6.16 (*S*1, SVM).

**Figure 6:**
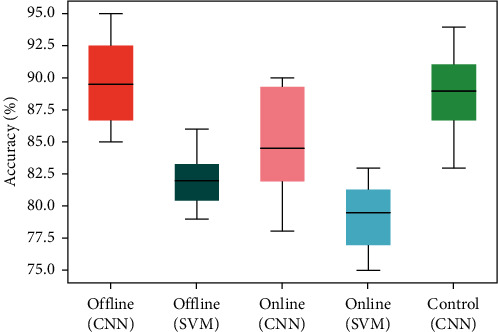
Accuracies of all subjects in each session. Note: each plot displays minimum, maximum, upper, medium, and lower quartiles.

The accuracies of all subjects in each session are displayed in Figure 6. The Lw-CNN's average offline classification accuracy was (90 ± 5)% and its median value was 89.75%. The SVM's average accuracy was (82.5 ± 3.5)% with a median of 82%. The mean online classification accuracy of Lw-CNN was (84 ± 6)%, wherein the medium was 85%. The average SVM value was (79 ± 4)% with a median value of 79.25%. In the control session, the recognition accuracy of the Lw-CNN model was (88.5 ± 5.5)% and its median value was 88.75%.

The effects of online and offline testing modes and classifiers on recognition accuracy were determined by conducting variance analysis on four groups of data of classification models, Lw-CNN and SVM offline and online, through two-way ANOVA. The classifiers exerted the most significant influence on recognition accuracy (*p* = 0.11*∗* 10 – 4, *η*^2^ = 0.447, *ω*^2^ = 0.426); different testing methods also significantly affected testing accuracy (*p* = 0.0045, *η*^2^ = 0.138, *ω*^2^ = 0.122). No significant influences were observed among different recognition modes or classifiers on recognition accuracy (*p* = 0.4177, *η*^2^ = 0.001, *ω*^2^ = −0.005). Though no significant difference between online and offline classification accuracy and real-time control accuracy was found in the Lw-CNN according to one-way ANOVA (*p* = 0.056), the real-time control and offline accuracy was obviously higher than the online accuracy (Figure 6).

**Figure 7:**
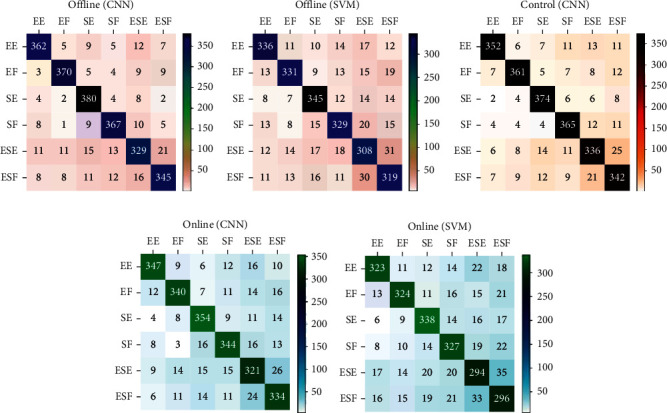
Confusion matrices obtained by different classifiers and different testing methods for eight subjects performing six motions: (a) offline testing results of Lw-CNN classifier; (b) offline testing results of SVM classifier; (c) real-time control testing results of Lw-CNN classifier; (d) online testing results of Lw-CNN classifier; (e) online testing results of SVM classifier.

Figure 7 shows confusion matrices of Lw-CNN and SVM classifiers in offline and online sessions as well as the control session experiment. Each confusion matrix synthesizes the recognition results of six motions across eight subjects. The Lw-CNN model performed the best in the offline testing scenario, showing more accurate recognitions of all six motions than other experimental modes or the SVM model. The quantity of accurate recognitions on the motions was higher in the offline mode than online regardless of which model was used. The quantities of wrong recognitions were higher for ESE and ESF motions than the other four motions.

**Figure 8:**
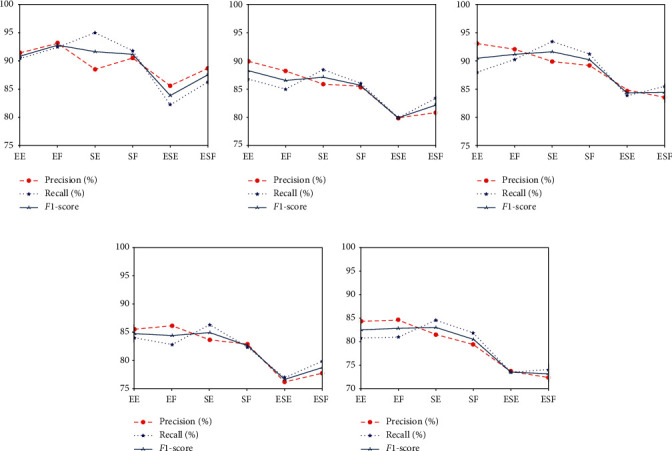
Precision, recall, and F1-score obtained by different classifiers and different testing methods. Each result curve combined recognition results of 6 motions of 8 subjects: (a) offline testing results of Lw-CNN classifier; (b) offline testing results of SVM classifier; (c) real-time control testing results of Lw-CNN classifier; (d) online testing results of Lw-CNN classifier; (e) online testing results of SVM classifier.

Figure 8 shows the precision, recall, and F1-score of recognition results of the six motions of the classifiers Lw-CNN and SVM in offline and online modes and the classifier Lw-CNN in the control session. The Lw-CNN model more accurately recognized all six motions than the SVM model. The recognition accuracy was also higher on all six motions in the offline testing mode than online. The recognition accuracy of the EF motion was highest in the offline mode (*p* = 92.5%); the recognition accuracy for the EE motion was highest in the online mode (*p* = 84%). The recognition results were worst for motions ESE and ESF regardless of which recognition model or testing mode was used. The confusion matrices and fold lines show lower recognition accuracies for SE and SF than EE or EF. The system appears to easily and accurately recognize elbow joint motions.

**Figure 9:**
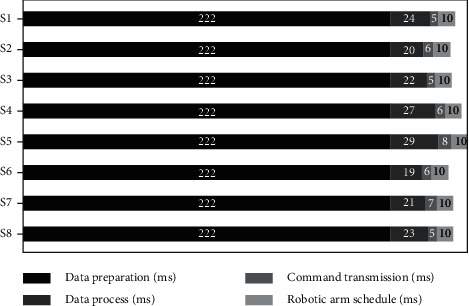
Statistical time-consumption results.

Figure 9 shows statistical results of time spent on each function of the system under real-time control testing with the proposed Lw-CNN model. The hardware delay caused by analog front end (AFE) circuit was neglected. Data transmission between the data collection card and PC was realized by a high-speed USB2.0 while time spent on FIFO function, transmission delay, and data preprocessing were also neglected. The response time of the whole system seriously influenced the sizes of analysis and slippage windows. Only three analysis windows were recognized while the window slippage rate was set to 15 ms to satisfy the real-time requirements, so preparation time for generating and inputting data to the Lw-CNN model was 192 + 15 + 15 = 222 ms. The computation time of the Lw-CNN model was dominated by the data processing time. The time spent sending control commands to the robotic arm after motion recognition via UART (115200 bps) was also counted. The robotic arm datasheet showed that the command response delay of robotic arm was 10 ms. The data processing time was 19–29 ms and the command transmission delay was 5–8 ms. The maximum total delay of the system was 269 ms (S5) and the minimum was 257 ms (S6), which satisfy the lowest relevant real-time requirements (300 ms delay).

## 4. Discussion

In this study, a three-channel sparse multichannel sEMG was used to collect sEMG signals of deltoid, biceps, and triceps of subjects' upper limbs in a motion intent recognition experiment centered on six shoulder and elbow joint motions. The motions of the robotic arm were controlled in real time using Lw-CNN classifiers. The Lw-CNN classifier performed better both online and offline than the SVM. Motion intent recognition via Lw-CNN is feasible in upper-limb rehabilitation training where feature values do not need to be selected manually; the optimum model parameters are instead obtained automatically by training the model on different subjects. The flexibility and robustness of the proposed model are stronger than those of traditional machine-learning methods.

Deep-learning models generally require more burdensome and lengthy computations than traditional machine-learning methods (e.g., SVM, LDA) [18], which is not conducive to real-time application [48]. The total time spent by the system in completing the recognition task should be lower than 300 ms to satisfy real-time requirements. A commercial robotic arm was used for end-to-end verification of the Lw-CNN model's real-time capability over an analysis window of 192 ms and window slippage rate of 15 ms. The real-time requirement was indeed satisfied in the experiment (<269 ms). The maximum time for recognition computation with the Lw-CNN model was 29 ms and the maximum computation time for recognition with the SVM classifier was 15 ms.

The real-time control accuracy of the proposed method was higher than its online classification accuracy, unlike the results of previous studies [33, 34]. This can be attributed to the hardware limits of the robotic arm. For example, when the robotic arm was under the EE state, the EF instruction was recognized as EE and triggered to move; this result was skipped, so it did not influence the overall accuracy. The subject was also allowed to see the motion results during the real-time control experiment, so he or she received real-time feedback from the robotic arm. The subject was asked to use a comfortable force level (preferably around 30–40% of maximum voluntary contraction), but he or she may have unconsciously made fine adjustments to the rate and force of the motions to realize higher accuracy. One-way ANOVA showed no significant difference (*p* = 0.056) in real-time control in offline versus online modes.

The recognition accuracies of Lw-CNN and SVM classification models on EE, EF, SE, and SF motions were higher than those on ESE or ESF (Figure 9). The ESE and ESF involved motions of both shoulder and elbow joints, so coordinated effects between muscles were generated and drove down the recognition accuracies of the classifiers. It is very difficult to recognize body motion intents in the context of coordinated muscle effect [19]. Additional sEMG or HD-sEMG channels and more complicated classification models are often used to increase classification accuracy in studies concerning gesture recognition [49]. However, this greatly increases the amount of system computation necessary making these methods unsuitable for real-time control.

The feasibility of sEMG signal-based motion recognition with deep models for the real-time control of upper-limb rehabilitation equipment was explored in this study. Relatively few channels were used with the Lw-CNN model to allow for effective real-time performance. The model showed high recognition rates on six separate motions. A deep model must be trained on a dataset composed of numerous samples; higher quantities of samples enhance the recognition accuracy. In this study, the analog signal was band-pass-filtered using AFE-board. In the data preprocessing, a digital band-pass filter and a 50 Hz notch were used to further improve the signal quality (SNR). For stroke patients, the muscle movement on the affected side was impaired, and the EMG signal strength was weaker compared to the healthy side; i.e., the signal SNR was relatively low. However, it is theoretically possible to identify the pathological signals using the proposed analog filtering and digital filtering preprocessing methods.

**Figure 10:**
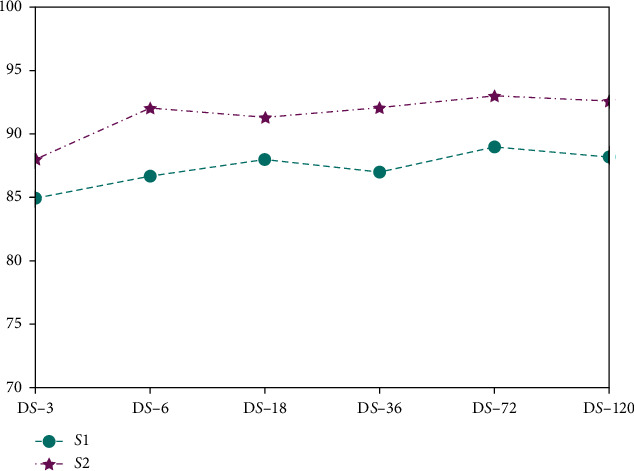
Offline testing with Lw-CNN model and different sizes of datasets (subjects 1 and 2).

In this study, 300 samples were selected for each subject to train Lw-CNN model. To determine whether greater quantities of samples would significantly enhance the model's accuracy, 2000 samples for each motion of subjects S1 and S2 were collected for a total of 12000 samples; 300, 600, 1800, 3600, 7200, and 12000 samples were then selected, respectively, to create training datasets (denoted DS-3, DS-6, DS-18, DS-36, DS-72, and DS-120).  Figure 10 shows the offline experimental results with the same Lw-CNN model and training parameters on these datasets. The recognition accuracy of S2 increased from 88% to 92% when the DS6 dataset was used. After further increase in the number of dataset samples, the accuracy did not substantially increase. The same phenomenon was observed for subject S1. When the DS-120 dataset was used, the recognition accuracy was slightly lower than that of the DS-72 dataset. Due to the large number of DS-120 dataset samples, the subject is required to make a lot of movements during the sample collection process, during which muscle fatigue and poor contact of the labeled electrodes were unavoidable. Some sample points might generate big noise, resulting in slight reduction of the model's recognition rate. As a whole, for the dataset of different scale sizes, the deep model had a higher recognition accuracy, showing the stronger generalization ability of the deep model.

Recognition accuracy is also influenced by analysis and slippage window sizes. Smaller windows and lower slippage rates enhance the real-time performance of the system and reduce the amount of recognition computation necessary but also significantly affect the recognition accuracy. The analysis window sizes selected in this study were 50 ms, 150 ms, and 192 m while slippage rates were 5 ms, 10 ms, and 15 ms. The corresponding recognition accuracies of the Lw-CNN model in offline mode are shown in Table 2. Recognition accuracy was markedly influenced by the window size and greatly reduced by lower slippage rates. When the 50 s analysis window was selected, the recognition accuracy was lower than 50%. Larger windows would be necessary in practice to satisfy real-time requirements.

Deep models were used in this study for high-precision recognition of elbow and shoulder joint motion intents in real time. The proposed method can be deployed in an intelligent man-machine interactive interface of upper-limb rehabilitation robots. It can also be used to improve the interactive flexibility of existing rehabilitation robots. The trained Lw-CNN model only has 367 k parameters and can be very easily deployed into an embedded system.

## 5. Conclusions

A novel technique was developed in this study to recognize upper-limb motion intents based on myoelectric patterns. The proposed technique can be used for upper-limb rehabilitation training based on the real-time control of robotic equipment. The offline and online classification accuracies of two classifiers, Lw-CNN and SVM, were measured on eight subjects. Both classifiers were more accurate when deployed offline than online. The Lw-CNN performed better than the SVM in online classification. The Lw-CNN was further used to recognize six separate upper-limb motions as a commercial robotic arm was controlled in real time. An average control accuracy of 88.75% was achieved. The proposed Lw-CNN model has only 367 K parameters. This paper concluded that applying a lightweight deep model for motion intent recognition was feasible and met the real-time requirements. The performance of the deep-learning model in the online control test and the evaluation criteria need to be further studied in the future. A real upper-limb rehabilitation robot will also be applied to optimize the rehabilitation motions discussed here. This will allow for the intelligent and flexible control of the upper-limb rehabilitation robot.

## Figures and Tables

**Table 1:** Subject features.

Subject ID	Age	Gender	Hand
*S*1	20	M	R
*S*2	21	F	R
*S*3	21	M	L
*S*4	19	M	R
*S*5	20	M	R
*S*6	22	M	R
*S*7	19	F	R
*S*8	20	F	L

**Table 2:** Offline testing accuracies with use of Lw-CNN model under different analysis window sizes and slippage rates.

Offline CNN-accuracy (%)									
	W50–O5^2^	W50–O10	W50–O15	W150–O5	W150–O10	W150–O15	W192–O5	W192–O10	W192–O15
*S*1	42	37	53	76	79	78	79	83	85
*S*2	32	41	40	79	82	84	81	84	88
*S*3	39	46	57	80	78	86	83	86	92
*S*4	22	37	30	83	85	89	85	87	91
*S*5	41	48	63	73	81	82	82	86	87
*S*6	43	40	45	80	90	93	89	90	94
*S*7	32	51	50	84	82	90	89	93	95
*S*8	29	38	48	80	82	87	84	86	86
Mean	35	42.25	48.25	79.38	82.38	86.13	84	86.88	89.75
SE^1^	7.45	5.39	10.22	3.54	3.74	4.76	3.59	3.23	3.77

^1^Standard error. ^2^w50–O5 denotes the analysis window of 50 ms, slippage 5 ms.

## Data Availability

The datasets generated during the current study are available from the corresponding author on reasonable request.
